# Supplementary material for: Gene therapy for aromatic L-amino acid decarboxylase deficiency by MR-guided direct delivery of AAV2-AADC to midbrain dopaminergic neurons
Source: Nat Commun. 2021 Jul 12;12:4251. doi: 10.1038/s41467-021-24524-8 (PMC8275582; doi:10.1038/s41467-021-24524-8)
Supplement: Supplementary file 1 — Supplementary Information [file 41467_2021_24524_MOESM1_ESM.pdf]

SUPPLEMENTARY INFORMATION

Supplementary Table 1. Raw Scores on Vineland Adaptive Behavior Scales – Second Edition (Vineland-2)

	Subject 2		Subject 3			Subject 4		Subject 5	
**Skill Area**	Month 12[#]	Month 24	BL	Month 12	Month 24	BL	Month 12	BL	Month 12
**Receptive Communication**	21	23	5	8	11	9	11	16	25
**Expressive Communication**	18	38	9	11	10	12	15	12	14
**Written Communication**	0	2	0	0	0	0	0	2	7
**Personal DLS**	4	9	0	5	5	0	4	4	8
**Domestic DLS**	0	0	0	0	0	0	0	0	0
**Community DLS**	2	5	0	0	1	0	0	1	1
**Interpersonal Relationships**	21	26	21	19	22	21	23	15	19
**Play & Leisure Time**	6	20	6	7	8	5	6	6	9
**Coping Skills**	10	17	4	4	4	3	5	4	4
**Gross Motor**	8	16	0	4	6	4	6	1	8
**Fine Motor**	10	17	0	0	1	6	9	1	12

DLS, Daily Living Skills
[#]BL assessment not performed
Note that data was not collected for Subject 1, and Subjects 6 and 7 are not included because they had not reached the Month 12 timepoint.

Supplementary Figure 1: FDOPA PET demonstrates increased AADC activity in midbrain and striatum 3 months after gene transfer.

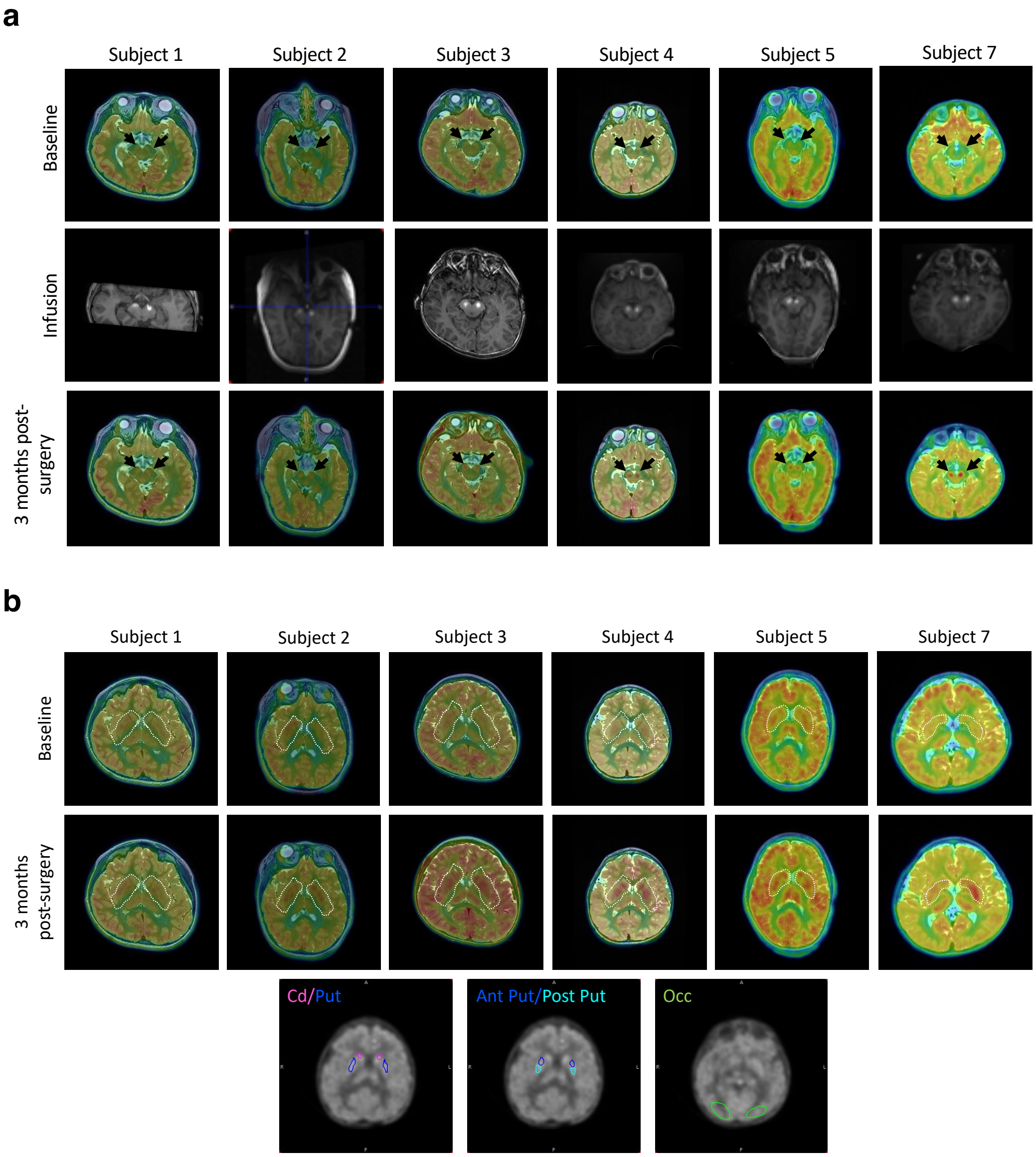

a, Images in the top row show FDOPA uptake in the midbrain before AAV2-hAADC infusion (baseline). The center row shows representative slices of the infusion at the midbrain for each subject as visualized by the MR-tracer (white regions in midbrain). Bottom row images present the FDOPA uptake in the same area of the midbrain 3 months after surgery. Black arrows in top and bottom rows point to the infusion target (SNc and VTA). **b,** Images in top and center rows show FDOPA uptake at the level of striatum. White dotted line indicates the striatum (caudate nucleus and putamen), the postsynaptic nucleus of the nigrostriatal pathway. Images in bottom row show representative slices used to select VOIs. Ki data were normalized by referencing them to the FDOPA uptake in the occipital lobe (VOI/Occ ratio).
