# Supplementary material for: Gene therapy for aromatic L-amino acid decarboxylase deficiency by MR-guided direct delivery of AAV2-AADC to midbrain dopaminergic neurons
Source: Nat Commun. 2021 Jul 12;12:4251. doi: 10.1038/s41467-021-24524-8 (PMC8275582; doi:10.1038/s41467-021-24524-8)
Supplement: Supplementary file 3 — Description of Additional Supplementary Files [file 41467_2021_24524_MOESM3_ESM.pdf]

Description of Additional Supplementary Files

Supplementary Movie 1:

Subject 2 (low-dose cohort) at Baseline (age 8.0 years) had audible stridor and severe hypokinesia. At Month 30 (00:20), she could stand and walk independently, and converse with her mother.

Supplementary Movie 2:

Subject 5 (high-dose cohort) at Baseline (age 6.7 years) had hypomimia, bradykinesia, marked axial hypotonia, and lower limb dystonia. At Month 18 (00:19) she could sit independently and communicate with an augmentative communication device, and at Month 21 she could rise from sitting to standing and take independent steps.

Supplementary Movie 3:

Subject 1 at Baseline (age 9 years) had akinesia and irritable mood. At Month 18, she had dramatic sustained improvement in mood and improved axial tone and head control. *Subject 3* (00:36) at Baseline (age 5.9 years) had severe hypotonia and akinesia. At Month 24, he had good head control and was able to sit independently on a stool. *Subject 4* (01:15) at Baseline (age 5.1 years) was able to roll but had severe hypotonia, poor head control, and irritable mood. At Month 18, there was marked improvement in muscle tone, and he was able to transition independently from supine to sitting and sit without support. *Subject 6* (01:53) at Baseline (age 6.8 years) had ptosis, marked hypotonia and hypokinesia. At Month 8, ptosis was much improved, and she was able to sit independently on the floor. There was subtle residual dyskinesia of the right hand. She also has stereotyped mouthing of the hands that we observed in all subjects after gene delivery, lasting for 6-24 months. *Subject 7* (02:31) at Baseline had hypotonia, severe bradykinesia, and a tracheostomy in place to maintain the upper airway. At Month 6, he had significantly improved axial tone and head control when supine and on pull to sit. (Subjects 1 and 3: low-dose cohort; Subjects 4,6, and 7: high-dose cohort).
