# Supplementary material for: Gene therapy for aromatic L-amino acid decarboxylase deficiency by MR-guided direct delivery of AAV2-AADC to midbrain dopaminergic neurons
Source: Nat Commun. 2021 Jul 12;12:4251. doi: 10.1038/s41467-021-24524-8 (PMC8275582; doi:10.1038/s41467-021-24524-8)
Supplement: Supplementary file 7 — Reporting Summary [file 41467_2021_24524_MOESM7_ESM.pdf]

nature research

Corresponding author(s): Krystof Bankiewicz

Last updated by author(s): Dec 17, 2020

Reporting Summary

Nature Research wishes to improve the reproducibility of the work that we publish. This form provides structure for consistency and transparency in reporting. For further information on Nature Research policies, see Authors & Referees and the Editorial Policy Checklist.

Please do not complete any field with "not applicable" or n/a. Refer to the help text for what text to use if an item is not relevant to your study.
For final submission: please carefully check your responses for accuracy; you will not be able to make changes later.

Statistics

For all statistical analyses, confirm that the following items are present in the figure legend, table legend, main text, or Methods section.

n/a	Confirmed	
☐	☒	The exact sample size (n) for each experimental group/condition, given as a discrete number and unit of measurement
☐	☒	A statement on whether measurements were taken from distinct samples or whether the same sample was measured repeatedly
☐	☒	The statistical test(s) used AND whether they are one- or two-sided<br>*Only common tests should be described solely by name; describe more complex techniques in the Methods section.*
☒	☐	A description of all covariates tested
☐	☒	A description of any assumptions or corrections, such as tests of normality and adjustment for multiple comparisons
☐	☒	A full description of the statistical parameters including central tendency (e.g. means) or other basic estimates (e.g. regression coefficient) AND variation (e.g. standard deviation) or associated estimates of uncertainty (e.g. confidence intervals)
☐	☒	For null hypothesis testing, the test statistic (e.g. F, t, r) with confidence intervals, effect sizes, degrees of freedom and P value noted<br>*Give P values as exact values whenever suitable.*
☒	☐	For Bayesian analysis, information on the choice of priors and Markov chain Monte Carlo settings
☒	☐	For hierarchical and complex designs, identification of the appropriate level for tests and full reporting of outcomes
☒	☐	Estimates of effect sizes (e.g. Cohen's d, Pearson's r), indicating how they were calculated

Our web collection on statistics for biologists contains articles on many of the points above.

Software and code

Policy information about availability of computer code

Data collection	iPlan v3.0
Data analysis	N/A

For manuscripts utilizing custom algorithms or software that are central to the research but not yet described in published literature, software must be made available to editors/reviewers. We strongly encourage code deposition in a community repository (e.g. GitHub). See the Nature Research guidelines for submitting code & software for further information.

Data

Policy information about availability of data

All manuscripts must include a data availability statement. This statement should provide the following information, where applicable:

- Accession codes, unique identifiers, or web links for publicly available datasets
- A list of figures that have associated raw data
- A description of any restrictions on data availability

The data that support the findings of this study are available from the corresponding author (K.B.) upon reasonable request.

Field-specific reporting

Please select the one below that is the best fit for your research. If you are not sure, read the appropriate sections before making your selection.

☒ Life sciences ☐ Behavioural & social sciences ☐ Ecological, evolutionary & environmental sciences

Life sciences study design

All studies must disclose on these points even when the disclosure is negative.

Sample size	Due to the ultra-rare nature of AADC deficiency (approx 135 patients worldwide), a sample size of 7 subjects was selected for this dose-escalation (Low dose n=3, High dose n=4) clinical trial
Data exclusions	Infusion coverage analysis: One data point was excluded due to perivascular leakage observed during the infusion.
Replication	A size of ≥n=3 per dosing group was used as replicates for experimental findings
Randomization	Randomization of participants does not apply to this study design since this is an open-label, dose-escalation study, and as such, participants were enrolled into the single arm of the study and dosed sequentially (Cohort 1 (n=3) - low dose) before increasing the dose.
Blinding	No blinding to treatment was applied to the study since it is a single-arm study for primarily assessing the safety of the first-in-human delivery of AAv2-AADC into the midbrain of patients with AADC deficiency. Motor function assessment (GMFM-88) was independently scored by two therapists, one who performed the test in-person, and a second therapist who reviewed each recorded session on video post-hoc and blinded to the timepoint of each assessment

Reporting for specific materials, systems and methods

We require information from authors about some types of materials, experimental systems and methods used in many studies. Here, indicate whether each material, system or method listed is relevant to your study. If you are not sure if a list item applies to your research, read the appropriate section before selecting a response.

Materials & experimental systems

n/a	Involved in the study
☒	☐ Antibodies
☒	☐ Eukaryotic cell lines
☒	☐ Palaeontology
☒	☐ Animals and other organisms
☐	☒ Human research participants
☐	☒ Clinical data

Methods

n/a	Involved in the study
☒	☐ ChIP-seq
☒	☐ Flow cytometry
☐	☒ MRI-based neuroimaging

Human research participants

Policy information about studies involving human research participants

Population characteristics	Male and female patients aged 4-9 years with a confirmed diagnosis of AADC deficiency by genetic testing and who met all inclusion/exclusion criteria were enrolled. The initial patients screened for this study had severe motor impairment and motor developmental delay despite treatment with currently available medications.<br>Subjects were enrolled sequentially into 2 dose groups: 3 subjects in Group 1 (8.3 x 10^11 vg/mL) followed by 4 subjects in Group 2 (2.6 x 10^12 vg/mL). The total infusate volume was 160 µL of vector for each subject.
Recruitment	Subjects were recruited via two patient foundations, the AADC Research Trust (UK) and the Pediatric Neurotransmitter Diseases (PND) Association (USA). Study information was made available on Clinicaltrials.gov and a study website at UCSF.<br>Subject 5, who had a milder phenotype, enrolled in Cohort 2 (higher dose) as parents decided to wait outcomes from Cohort 1. While Subject 5 milder condition could impact the outcome of receiving a higher dose, Subject 2 was of similar severity at baseline and shows similar improvements to those reported for Subject 2, despite receiving the lower dose. Based on the data collected to date, we have not observed a dose-response between the 2 doses tested.
Ethics oversight	IRB at University of California San Francisco (Approval No.: 15-17756) and The Ohio State University (Approval No.: 2018H269). Informed consent and consent to publish was obtained from the legally authorized representative of the participants.

Note that full information on the approval of the study protocol must also be provided in the manuscript.

Clinical data

Policy information about clinical studies

All manuscripts should comply with the ICMJE guidelines for publication of clinical research and a completed CONSORT checklist must be included with all submissions.

Clinical trial registration	NCT02852213
Study protocol	Clinical protocol can be accessed in ClinicalTrials.gov
Data collection	The Study was conducted at the UCSF Benioff Children's Hospital in San Francisco, CA and at The Ohio State University Wexner

Data collection	Medical Center in Columbus, OH. Recruitment was performed by invitation and in collaboration with the AADC Research Trust. Participants and their families stayed locally in either San Francisco and Columbus for 3 months after surgery for close post-surgery monitoring and returned for follow-up visits at 6, 12, 18 and 24 months.
Outcomes	Primary Outcome Measures :<br>Safety [Time Frame: 2 years]<br>Assessment of adverse events related to surgery (including intracerebral hemorrhage or stroke, CNS infection) and gene transfer (including severity of post-operative dyskinesia)<br><br>Efficacy [Time Frame: 1 year]<br>Change in CSF neurotransmitter metabolite concentrations after gene transfer (increase in homovanillic acid (HVA) and 5-hydroxyindoleacetic acid (5-HIAA), and elevated 3-O-methyldopa (3-OMD) concentrations)<br><br>Secondary Outcome Measures :<br>Gross Motor Function Measure [Time Frame: 2 years]<br>Increase in Gross Motor Function Measure-88 (GMFM-88) score<br><br>Symptom Log [Time Frame: 1 year]<br>Change in frequency and severity of oculogyric crises<br><br>Fluorodopa PET scan [Time Frame: Evaluated at 3 months and 2 years]<br>Increase in signal in the striatum on FDOPA-PET imaging as brain AADC activity measure

Magnetic resonance imaging

Experimental design

Design type	Real-time MR imaging of brain infusion of AAV gene product admixed with MR tracer (gadoteridol)
Design specifications	MR imaging was used to confirm targeting, and monitor infusion distribution rather than as a separate data endpoint
Behavioral performance measures	N/A

Acquisition

Imaging type(s)	Weighted T1
Field strength	3T
Sequence & imaging parameters	*Specify the pulse sequence type (gradient echo, spin echo, etc.), imaging type (EPI, spiral, etc.), field of view, matrix size, slice thickness, orientation and TE/TR/flip angle.*
Area of acquisition	Whole brain

Diffusion MRI ☐ Used ☒ Not used

Preprocessing

Preprocessing software	N/A
Normalization	N/A
Normalization template	N/A
Noise and artifact removal	N/A
Volume censoring	N/A

Statistical modeling & inference

Model type and settings	N/A
Effect(s) tested	*Define precise effect in terms of the task or stimulus conditions instead of psychological concepts and indicate whether ANOVA or factorial designs were used.*

Specify type of analysis: ☐ Whole brain ☐ ROI-based ☐ Both

Statistic type for inference (See Eklund et al. 2016)	N/A
Correction	N/A

Models & analysis

n/a	Involved in the study
☒	☐ Functional and/or effective connectivity
☒	☐ Graph analysis
☒	☐ Multivariate modeling or predictive analysis

This checklist template is licensed under a Creative Commons Attribution 4.0 International License, which permits use, sharing, adaptation, distribution and reproduction in any medium or format, as long as you give appropriate credit to the original author(s) and the source, provide a link to the Creative Commons license, and indicate if changes were made. The images or other third party material in this article are included in the article's Creative Commons license, unless indicated otherwise in a credit line to the material. If material is not included in the article's Creative Commons license and your intended use is not permitted by statutory regulation or exceeds the permitted use, you will need to obtain permission directly from the copyright holder. To view a copy of this license, visit http://creativecommons.org/licenses/by/4.0/
